# Intraoral Scanners for Three-Dimensional Assessment of Peri-Implant Soft Tissues: A Systematic Review of Clinical Studies

**DOI:** 10.4317/jced.64074

**Published:** 2026-05-29

**Authors:** Kakhramon Shomurodov, Rano Mirkhusanova, Elyor Bekmurodov, Oybek Idiev

**Affiliations:** 1 Department of Maxillofacial Surgery, Faculty of Dentistry, Tashkent State Medical University, Tashkent, Uzbekistan; 2 Department of Prosthodontics, Faculty of Dentistry, Bukhara State Medical Institute named after Abu Ali ibn Sino, Bukhara, Uzbekistan

## Abstract

**Background:**

Precise assessment and longitudinal monitoring of peri-implant soft tissues are essential in implant dentistry, and digital workflows may enable objective and reproducible quantification of soft-tissue dynamics. Objectives: To systematically evaluate the clinical performance of intraoral scanners (IOSs) for the three-dimensional (3D) assessment of peri-implant soft tissues, focusing on accuracy, reliability, reproducibility, and clinical applicability.

**Material and Methods:**

This systematic review followed PRISMA guidelines and was registered in PROSPERO (CRD420261295073). Electronic searches of PubMed, Scopus, and Web of Science were conducted up to December 2025. Clinical studies evaluating IOS-based 3D assessment of peri-implant soft tissues were included. Data extraction and risk-of-bias assessment (ROBINS-I) were performed independently by two reviewers.

**Results:**

Five clinical studies involving 56 patients and 62 implants met the inclusion criteria. IOS-based digital workflows enabled precise the 3D quantification of peri-implant soft-tissue morphology, including contour replication and volumetric changes. The removal of provisional restorations resulted in immediate mucosal collapse and increased dimensional deviation, highlighting the importance of tissue stabilization during digital acquisition. Digital morphometric analysis demonstrated high repeatability and reproducibility, with low measurement variability. Compared with conventional clinical methods, IOSs provided greater sensitivity for detecting subtle soft-tissue changes and enabled objective longitudinal monitoring. The overall risk of bias was moderate, mainly due to small sample sizes and pilot study designs.

**Conclusions:**

The available evidence on the use of IOS for the 3D assessment of peri-implant soft tissues is limited and primarily derived from small pilot studies with a moderate risk of bias. While the initial findings indicate the potential to detect morphological and volumetric changes, the reliability and clinical relevance of these measurements remain uncertain. Further well-designed, adequately powered longitudinal studies with standardized protocols are required to clarify their validity and clinical applicability.

## Introduction

Peri-implant soft tissues are one of the key factors of the long-term success, biological stability, and aesthetic integration of dental implants. Beyond osseointegration, the morphology, thickness, and volumetric stability of the peri-implant mucosa are critical determinants of peri-implant health, resistance to recession, and patient-reported aesthetic satisfaction. Clinical and experimental evidence demonstrates that the soft-tissue phenotype, keratinized mucosa width, and mucosal thickness are closely linked to peri-implant tissue stability and the prevention of peri-implant diseases (1 , 2). Consequently, precise assessment and longitudinal monitoring of peri-implant soft tissues have become fundamental components of contemporary implant dentistry. The evaluation of peri-implant soft tissue has traditionally relied on periodontal probing, clinical indices, photography, and radiographic examination. While these methods provide valuable clinical information, the measurements are often operator-dependent, invasive, and limited in their ability to detect subtle three-dimensional (3D) morphological changes. Radiographic imaging remains the gold standard for hard tissue evaluation but offers limited visualization of peri-implant soft tissues (3). Furthermore, two-dimensional (2D) documentation cannot adequately capture volumetric alterations or surface contour dynamics, which are essential for understanding peri-implant tissue remodelling and aesthetic outcomes. The rapid advancement of digital dentistry has introduced intraoral scanners (IOSs) as powerful tools for capturing highly accurate 3D representations of intraoral structures. Using digital surface mapping, superimposition techniques, and volumetric analysis, IOSs enable clinicians and researchers to monitor soft-tissue dimensional changes over time with high precision (4 , 5). An increasing number of clinical studies have applied IOS-based methodologies to assess peri-implant soft tissue thickness, contour stability, volumetric alterations, and mucosal recession. Digital workflows offer objective and reproducible quantification of soft-tissue dynamics and may improve the reliability of longitudinal assessment compared with conventional techniques (6). Moreover, IOS-derived data can facilitate digital treatment planning, outcome prediction, and individualized implant therapy. Despite growing clinical adoption, substantial heterogeneity exists among studies regarding scanning protocols, acquisition accuracy, software processing, and outcome reporting. Uncertainty remains concerning the accuracy, reproducibility, and overall clinical applicability of IOSs for 3D peri-implant soft-tissue evaluation (7). To date, no comprehensive synthesis has systematically evaluated the clinical performance of IOSs specifically for the 3D assessment of peri-implant soft tissues. A critical appraisal of available clinical evidence is required to clarify the reliability of IOSs and their clinical role. Such analysis is essential for establishing standardized digital assessment protocols and guiding future investigations in peri-implant tissue monitoring. Therefore, the systematic review aims to evaluate the clinical performance of IOSs in the 3D assessment of peri-implant soft tissues, with particular emphasis on accuracy, reproducibility, reliability, and clinical applicability in monitoring morphological and volumetric soft tissue changes.

## Material and Methods

This systematic review was conducted in accordance with the PRISMA guidelines (8) and was prospectively registered in the PROSPERO database (registration ID: CRD420261295073) to ensure methodological transparency and reproducibility. The review aimed to evaluate the effectiveness of 3D digital assessment methods for evaluating peri-implant soft-tissue changes using IOSs. No review protocol deviations occurred after PROSPERO registration. Search Strategy: A comprehensive electronic search was conducted using the PubMed, Scopus, and Web of Science databases to identify relevant studies. Table 1 presents the advanced search strings for each database.

Table 1

The search was limited to original research articles published in English involving human subjects. Studies focusing solely on in vitro models or animal data or not reporting diagnostic performance outcomes were excluded. The initial electronic search was conducted on November 2, 2025. The search was last updated on December 24, 2025. Additional relevant studies were identified through manual screening of reference lists from the included articles. Inclusion and exclusion criteria: The inclusion criteria for this systematic review were defined using the PICOS framework. The population (P) comprised adults undergoing dental implant therapy with reported peri-implant soft tissue outcomes. The intervention (I) included the assessment of peri-implant soft tissues using IOSs, including digital impressions, optical impressions, and volumetric and linear soft-tissue measurements derived from IOS data. Studies were eligible regardless of whether they compared (C) digital assessment tools with conventional diagnostic approaches or other diagnostic devices or included no comparator. The outcomes (O) of interest were diagnostic accuracy parameters (trueness, precision, validity), repeatability/reproducibility, measurement error, agreement with reference standards, and linear or volumetric peri-implant soft tissue changes. Eligible study designs (S) included original diagnostic accuracy studies, both retrospective and prospective, reporting quantitative results. Only full-text articles published in English were included. Exclusion criteria were studies not involving IOS for peri-implant tissue evaluation, studies lacking diagnostic performance outcomes, studies reporting the digital evaluation of soft-tissue parameters not related to implants, case reports, review articles, editorials, conference abstracts, expert opinions, non-human or in vitro studies, technical notes, and publications without accessible full text. Study selection: All identified references were imported into EndNote software (version X20; Clarivate Analytics, Philadelphia, USA) to identify and remove duplicate records. The reviewers independently screened the titles and abstracts of the retrieved articles for relevance using Rayyan QCRI (Rayyan, Doha, Qatar). Full-text articles deemed potentially eligible were retrieved and assessed in detail for inclusion according to the predefined eligibility criteria. Any disagreements during the selection process were resolved through discussion and consensus between the reviewers. Data extraction: The two reviewers (K.Sh. and R.M.) independently extracted data from the full-text versions of all eligible articles using a standardized approach to ensure accuracy and consistency. The following data were extracted from each study: first author and publication year, study design, sample size (patients/implants), implant location, intervention, type of IOS and digital analysis software, assessed outcomes, any comparator, key findings, and limitations (Table 2).

Table 2

Any discrepancies or uncertainties encountered during the extraction process were resolved through collaborative discussion among all authors to ensure data reliability and methodological rigour. Risk of bias and quality assessment: The risk of bias and applicability concerns were assessed independently by two reviewers (K.Sh. and R.S.) using the ROBINS-I tool. The evaluation was performed across the following domains: bias due to confounding, participant selection, classification of interventions, deviations from intended interventions, missing data, measurement of outcomes, and selection of the reported result. Each domain was judged as having a low, moderate, serious, or critical risk of bias according to ROBINS-I guidance. Disagreements were resolved by consensus among the authors. Domain-level judgements were supported by explicit justifications based on study-specific characteristics (e.g., lack of control for confounding variables, unclear inclusion criteria, incomplete follow-up, and absence of blinding in outcome assessment) to enhance transparency. An overall risk-of-bias judgement for each study was assigned according to the highest level of bias observed across domains, consistent with ROBINS-I recommendations. Due to substantial clinical and methodological heterogeneity among the included studies, a quantitative meta-analysis was not considered appropriate; therefore, a narrative synthesis was undertaken. The study designs were predominantly small-scale pilot or exploratory clinical studies with limited sample sizes (6-20 participants), which reduced statistical comparability and increased imprecision. Some investigations reported linear or volumetric deviations in micrometres or millimetres, while others used root mean square (RMS) values, dimensional width changes, or volumetric healing metrics at different anatomical locations and time points. Thus, no statistical pooling was performed, and heterogeneity was not formally assessed (e.g., using the I² statistic). Outcome measures were not standardized across studies. Given the narrative approach and the clinical and methodological heterogeneity of the included studies, no formal assessment of publication bias (e.g., funnel plots or Egger's test) or certainty of evidence (e.g., GRADE) was conducted.

## Results

**Figure 1:**
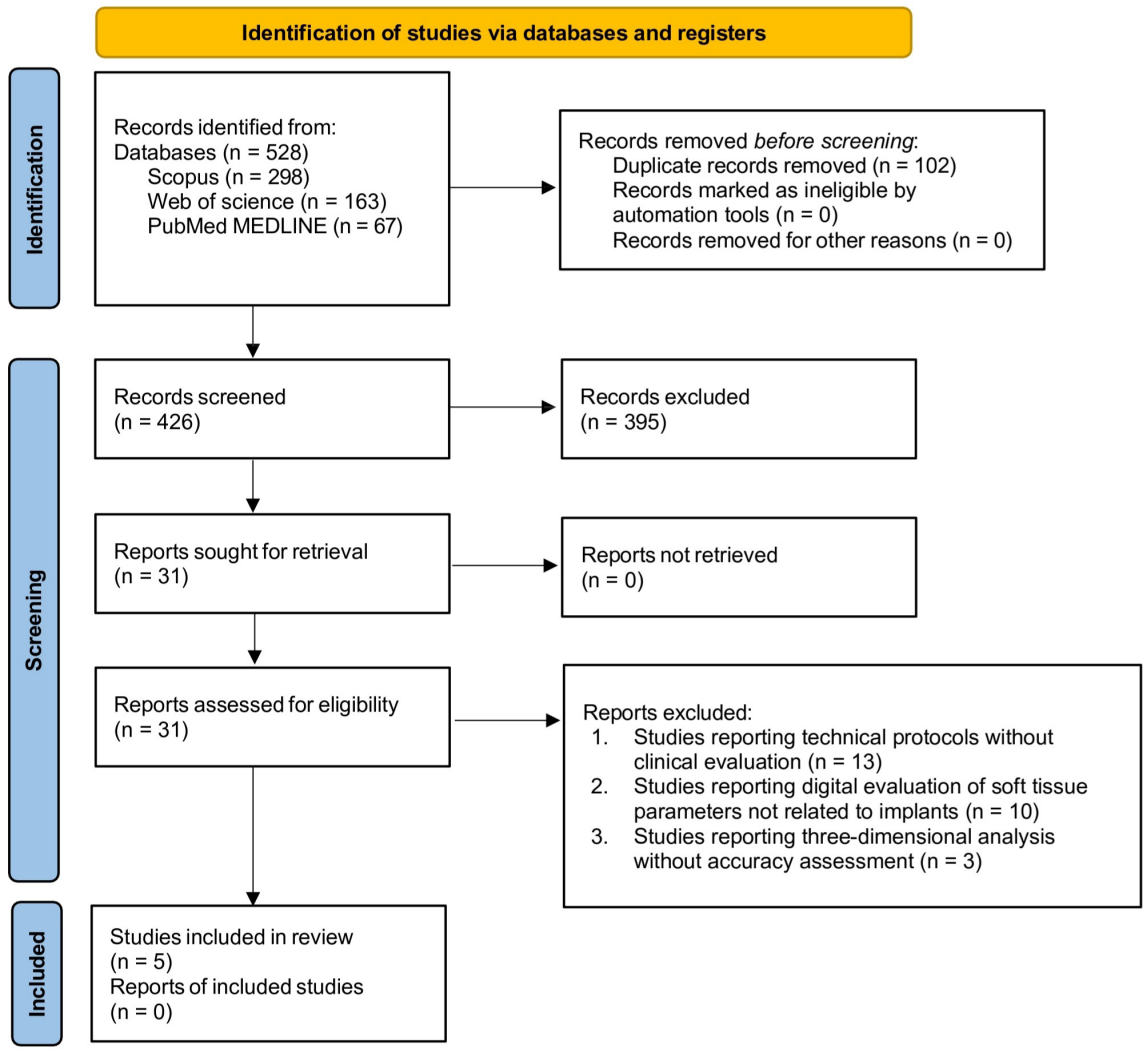
PRISMA flow diagram.

Search results: A total of 528 records were identified through database searches. After removing 102 duplicates, 426 records were screened, and 395 were excluded based on titles and abstracts. Five studies met the inclusion criteria and were included in the qualitative synthesis and were included in the final qualitative synthesis, as shown in the PRISMA flow diagram (Fig. 1).

1

Study characteristics: The studies included in this systematic review involved 3D assessment of peri-implant soft tissues using intraoral scanning combined with digital superimposition and morphometric analysis. Across investigations, outcomes were primarily quantitative and focused on dimensional, volumetric, and morphological evaluation of peri-implant mucosa. Four studies assessed dimensional accuracy and soft-tissue contour replication using STL superimposition of digital models (9 - 12). The clinical study by Xiong et al. quantitatively evaluated the influence of implant-supported provisional restorations on the accuracy of peri-implant mucosal replication using 2D and 3D deviation analysis (9). Similarly, Ling et al. measured dimensional and volumetric deviations of peri-implant mucosa after definitive crown delivery, including labial and palatal soft-tissue thickness, volumetric changes, and aesthetic outcomes assessed by the Pink Esthetic Score (PES) (12). The crossover clinical study by Silva Marques et al. compared the accuracy of 3D soft-tissue replication of digital and conventional impressions, using RMS deviation and effect size calculations to quantify differences in soft-tissue emergence profiles (10). In contrast, the pilot study by Duran et al. focused on dimensional variations in peri-implant soft tissue profile immediately after removal of interim restorations, measuring buccolingual and mesiodistal linear deviations following digital model superimposition (11). One study primarily evaluated volumetric soft-tissue healing dynamics using a novel digital morphometric technique. Docampo-Vázquez et al. performed longitudinal 3D volumetric analysis of free gingival graft healing around implants, assessing tissue volume changes over multiple time points and analysing measurement repeatability and reproducibility using Gage R&amp;R statistics (13). Overall, qualitative synthesis demonstrated that IOS-based digital workflows consistently enabled precise 3D quantification of peri-implant soft-tissue morphology, including static contour replication and dynamic volumetric changes during healing and prosthetic phases. Table 2 summarizes the study characteristics and outcome measures. Risk of bias and quality assessment: The risk of bias and applicability concerns were assessed using the ROBINS-I tool. The overall methodological quality was acceptable: three studies (9 , 10 , 12) showed moderate risk of bias, one (13) moderate-to-serious risk, and one (11) serious risk. The most frequent concern was confounding, mainly due to small sample sizes, pilot designs, and limited control of variables such as the soft-tissue phenotype, implant position, prosthetic conditioning, and healing dynamics. These factors increased the susceptibility to residual confounding, particularly in non-comparative studies, with the highest concerns observed in the pilot study by Duran et al. (11) and the exploratory study by Docampo-Vázquez et al. (13). Selection bias was generally low to moderate, although small sample sizes reduced external validity, as illustrated by the crossover study of Silva Marques et al. (10). Bias related to intervention classification, deviations from intended interventions, and missing data were low due to clearly described and standardized digital workflows reported by Xiong et al. (9) and Ling et al. (12). Measurement bias was judged moderate, as assessor blinding and operator calibration were inconsistently reported despite the use of validated digital morphometric methods, although repeatability testing was performed in the study by Docampo-Vázquez et al. (13). The selective reporting risk was low across studies. Overall, the evidence was judged to be of moderate methodological quality, primarily limited by small sample sizes, pilot study designs, and potential confounding. Table 3 presents detailed risk-of-bias assessment.

Table 3

## Discussion

This systematic review synthesized evidence from five original investigations evaluating the clinical performance of IOSs for 3D assessment of peri-implant soft tissues. Overall, the included studies indicated that IOSs enable reproducible 3D quantification of peri-implant soft tissue morphology. Accurate replication of peri-implant soft-tissue morphology is essential for achieving predictable aesthetic and functional outcomes in implant therapy. Across the included clinical studies, IOSs demonstrated the ability to reproduce peri-implant soft tissue morphology with clinically acceptable accuracy; however, replication accuracy was strongly influenced by soft-tissue support and acquisition protocol. The clinical study by Xiong et al. showed that removal of the implant-supported provisional restoration (ISPR) resulted in immediate peri-implant mucosal collapse within 20 s, producing approximately 500 µm greater dimensional deviation compared with scans obtained with the ISPR in place. The deformation primarily affected the cuff-like submucosal region, which is biomechanically less stable and prone to collapse after the loss of physical support (9). These findings indicate that the presence of a provisional restoration is critical for maintaining peri-implant mucosal architecture and ensuring accurate digital replication. Consistent with these observations, Duran et al. reported dimensional deviations of 0.51-1.35 mm in the peri-implant emergence profile following the removal of interim restorations, confirming that soft-tissue collapse substantially compromises the accuracy of direct digital acquisition (11). Similarly, Ling et al. demonstrated that peri-implant soft tissue undergoes significant collapse after provisional crown removal, leading to decreased labial and palatal mucosal height and altered tissue thickness. This deformation resulted in deviation of the subgingival contour and reduced accuracy of direct digital scanning. The authors further showed that the indirect digital technique, which captures the emergence profile of the provisional crown rather than the collapsed mucosa, produced significantly smaller soft-tissue deviations and more accurate replication of peri-implant contours (12). The crossover clinical study by Silva Marques et al. also confirmed that intraoral scanning enables quantitative 3D evaluation of peri-implant soft tissue replication. The reported overall RMS discrepancy for peri-implant soft tissues was 243.9 µm, representing a statistically significant difference compared with surrounding natural soft tissues; however, deviations remained below the clinically detectable threshold of approximately 1 mm. Importantly, the study showed that digital superimposition techniques provide high sensitivity for detecting peri-implant soft-tissue discrepancies and may prevent approximately 200 µm of soft-tissue replication error when using optimized acquisition protocols (10). Although the primary focus of Docampo-Vázquez et al. was volumetric soft-tissue healing, their findings support the reliability of digital morphometric analysis for capturing peri-implant soft-tissue morphology with high repeatability and measurement sensitivity (13). A key advantage of IOS-based digital workflows is the ability to quantify volumetric and morphological soft-tissue changes over time. The included studies demonstrated that digital superimposition techniques can detect small but significant changes in peri-implant mucosal volume and contour during healing, graft maturation, and prosthetic conditioning (12 , 13). Volumetric analysis revealed measurable tissue remodelling both immediately after surgical or prosthetic intervention and throughout longitudinal follow-up periods (13). These findings confirm that IOSs enable dynamic monitoring of peri-implant soft tissue behaviour, including tissue collapse after provisional restoration removal and gradual volumetric stabilization during healing (12). Digital morphometric techniques were particularly sensitive in detecting early dimensional changes that may not be clinically visible, suggesting potential value for the early identification of peri-implant soft tissue instability. Such capability may be clinically relevant for optimizing soft-tissue augmentation procedures, guiding provisional restoration design, and improving long-term peri-implant aesthetic outcomes. Furthermore, digital volumetric monitoring provides a quantitative alternative to traditional clinical measurements, which are often limited to linear probing or 2D evaluation. By enabling 3D visualization of tissue remodelling, IOSs may enhance the understanding of peri-implant soft tissue biology and support individualized treatment planning (13). The included studies indicate that IOS-based morphometric analysis generally yields reliable and reproducible measurements, although methodological variability across studies warrants cautious interpretation. The controlled intra-subject clinical study by Xiong et al. indicated that standardized acquisition and superimposition protocols enable consistent, repeatable quantification of peri-implant soft-tissue deviation, supporting the technical reliability of IOS-based morphometric evaluation (9). Strong methodological evidence for reproducibility was provided by Docampo-Vázquez et al., who performed a Gage R&amp;R statistical analysis to evaluate their digital volumetric measurement technique. The reported measurement system variability was low (0.6% within-operator and 7.6% between-operator), confirming that the digital morphometric approach provides accurate, repeatable, and reproducible measurements, with most variability reflecting true biological tissue changes rather than measurement errors (13). Similarly, Silva Marques et al. validated the reliability of reverse-engineering software through repeated dataset superimposition, highlighting stable measurement performance across multiple repetitions and confirming the ability of digital workflows to consistently detect peri-implant soft-tissue deviations (10). The use of standardized alignment procedures and software-based trueness validation contributed to improved measurement consistency and reduced operator-dependent variability. Evidence from Ling et al. further supports reproducible digital assessment, as standardized intraoral scanning protocols and best-fit superimposition allowed consistent comparison of direct and indirect digital acquisition techniques across time points (12). Likewise, Duran et al. demonstrated that automated digital superimposition using stable reference structures enables reproducible measurement of dimensional changes in peri-implant soft-tissue, although unsupported soft tissues were shown to be inherently unstable and may introduce biological variability independent of measurement precision (11). Traditional clinical assessment of peri-implant soft tissues relies primarily on visual inspection, periodontal probing, photographs, and customized impression techniques, which are inherently operator-dependent and often limited to 2D or linear measurements. Conversely, digital morphometric analysis enables objective 3D quantification of soft tissue contour and volumetric changes, offering improved detection of subtle morphological variations. Xiong et al. directly compared intraoral scanning with conventional impressions and showed that both methods can replicate peri-implant soft-tissue profiles. However, conventional impressions using the provisional restoration as an impression coping served as a stable reference model for digital superimposition and revealed that unsupported mucosa captured by routine intraoral scanning may deform rapidly after provisional removal (9). Similarly, Silva Marques et al. reported that intraoral scanning achieved comparable trueness but detected statistically significant differences in peri-implant soft-tissue replication. Importantly, digital superimposition showed higher sensitivity than visual and clinical methods, enabling the detection of soft-tissue variations below clinically perceptible thresholds (10). Ling et al. further emphasized that conventional duplication of the provisional emergence profile remains a reliable reference for transferring peri-implant tissue contours, whereas direct digital acquisition may be affected by soft-tissue collapse. Their findings highlighted that an indirect digital technique - replicating the provisional crown contour rather than the unsupported mucosa - reduced soft-tissue deviation and improved contour duplication compared with direct digital scanning (12). The pilot study by Duran et al. highlighted the limitations of digital impressions when capturing unsupported peri-implant soft tissues immediately after the removal of interim restorations. Significant discrepancies were observed between the emergence profile recorded using conventional impression techniques and the digitally captured profile, primarily due to rapid soft-tissue collapse (11). This reinforces the importance of tissue stabilization and appropriate acquisition protocols when using digital methods. Conversely, Docampo-Vázquez et al. demonstrated that digital morphometric techniques enable objective, repeatable volumetric measurement of soft-tissue healing and provide reliability comparable to conventional clinical measurement methods, while offering superior capability for 3D analysis and longitudinal monitoring (13). Overall, the evidence suggests that intraoral scanning does not replace conventional assessment but extends diagnostic capability by enabling objective, 3D, and highly sensitive evaluation of peri-implant soft tissues. Conventional methods remain valuable for stable tissue transfer and routine clinical evaluation, whereas digital workflows provide enhanced precision for morphometric analysis and longitudinal monitoring. The optimal clinical approach may therefore involve the integration of both methods, supported by standardized acquisition protocols to minimize soft-tissue deformation and measurement variability. This systematic review has several limitations. First, the number of eligible clinical studies was limited, reflecting the emerging nature of digital morphometric assessment in implant dentistry. Second, most included studies were pilot or exploratory investigations with small sample sizes, reducing statistical power and generalizability. Third, heterogeneity in study design, scanning protocols, and outcome measures limited direct comparison and prevented quantitative synthesis. Fourth, long-term data on peri-implant soft-tissue stability obtained from IOSs are currently lacking. Finally, potential publication bias cannot be excluded, as studies reporting negative findings may be underrepresented.

## Conclusions

The available evidence on IOS use for 3D assessment of peri-implant soft tissues is limited and primarily derived from small pilot studies with a moderate risk of bias. While preliminary findings suggest the potential to detect morphological and volumetric changes, the reliability and clinical relevance of these measurements remain uncertain, and no definitive conclusions can be drawn. Further high-quality clinical research is required to strengthen the evidence base, including adequately powered prospective studies with standardized digital acquisition and analysis protocols. Methodological consistency, as well as calibration and validation of morphometric approaches, is required to improve comparability across studies. Additionally, the establishment of clinically meaningful thresholds for soft tissue changes and the evaluation of long-term peri-implant tissue stability using digital methods warrant further investigation. The role of emerging approaches, including automated analysis techniques, remains to be determined.

## Figures and Tables

**Table 1:** Table Search strategy.

Databases	Search Strategy
PubMed	(("Dental Implants"[Mesh] OR implant*[tiab] OR peri-implant*[tiab]) AND ("Intraoral Scanners"[Mesh] OR "intraoral scanner*"[tiab] OR "optical impression*"[tiab] OR "digital impression*"[tiab] OR IOS[tiab]) AND ("Soft Tissue"[Mesh] OR "gingival tissue*"[tiab] OR gingiva*[tiab] OR mucosa*[tiab] OR "peri-implant soft tissue*"[tiab] OR "emergence profile"[tiab]) AND ("Diagnostic Accuracy"[Mesh] OR accuracy[tiab] OR validity[tiab] OR precision[tiab] OR reproducibility[tiab] OR reliability[tiab] OR trueness[tiab] OR repeatability[tiab]))
Scopus	TITLE-ABS-KEY ((implant* OR peri-implant*) AND ("intraoral scanner*" OR "digital impression*" OR "optical impression*" OR IOS) AND ("soft tissue*" OR gingiva* OR mucosa* OR "peri-implant soft tissue*" OR "emergence profile") AND (accuracy OR validity OR precision OR reliability OR reproducibility OR trueness OR repeatability))
Web of Science	TS=((implant* OR peri-implant*) AND ("intraoral scanner*" OR "digital impression*" OR "optical impression*" OR IOS) AND ("soft tissue*" OR gingiva* OR mucosa* OR "peri-implant soft tissue*" OR "emergence profile") AND (accuracy OR validity OR precision OR reliability OR reproducibility OR trueness OR repeatability))

1

**Table 2:** Table Characteristics of included studies.

Study ID and design	Participants / Implants with Location,Intervention	IOS system and digital analysis software	Outcomes assessed and Comparator	Major findings	Limitations
Xiong et al. [9] prospective clinical study	16 / 16 anterior maxillaIntervention: IOS of peri-implant soft tissue with and without implant-supported provisional restoration (ISPR)	TRIOS (3Shape, Copenhagen, Denmark)Software: Geomagic Control X (3D Systems, Rock Hill, USA)	Peri-implant soft tissue deviation (µm) in 2D and 3D; accuracy of mucosal profile replicationComparator: conventional impression using ISPR as impression coping	Removal of ISPR led to rapid peri-implant mucosal collapse. IOS with ISPR in place showed significantly higher accuracy than IOS without ISPR (mean 3D deviation: 230.6 ± 85.5 μm vs 414.7 ± 116.0 μm; p < .0001)	Small sample size; limited to single implants on only anterior maxilla; short-term assessment; conventional impression may introduce material-related inaccuracies
da Silva Marques et al. [10] cross-over pilot clinical study	6 / 6 anterior maxillaIntervention: digital intraoral impression obtained immediately after removal of provisional crown and compared with conventional impression using customized implant impression coping (CIIC)	TRIOS (3Shape, Copenhagen, Denmark)Software: Geomagic Design X (3D Systems, Rock Hill, USA)	3D trueness (RMS, μm) of hard and soft tissues, peri-implant emergence profile, effect size (Hedges’ g)Comparator: conventional polyvinyl siloxane impression with customized implant impression coping and extraoral scanning	Digital impressions showed statistically significant differences in peri-implant soft tissue replication compared with CIIC (overall RMS ≈ 244 μm; large effect size), but discrepancies were below the clinically detectable threshold (1 mm)	Small sample size; pilot design; limited to single implants on only anterior maxilla; no correlation analysis for implant depth, connection type, or gingival biotype; clinical impact on esthetic outcomes not assessed
Duran et al. [11] pilot clinical study	10 / 10 anterior maxillaIntervention: digital intraoral impression of peri-implant soft tissue immediately after removal of implant-supported fixed interim restoration (ISFIR); STL superimposition and linear cross-sectional measurements	CEREC Omnicam (Sirona Dental Systems, Bensheim, Germany)Software: Cerec SW 4.4 (SironaDental System, Bensheim, Germany)	Linear dimensional changes (mm) of peri-implant soft tissue profile in buccolingual and mesiodistal directions at coronal and mid-gingival levelsComparator: conventional polyvinyl siloxane impression using ISFIR as customized impression transfer, followed by stone cast fabrication and digital scanning	Significant dimensional discrepancies were observed between the ISFIR emergence profile and unsupported peri-implant soft tissue captured by IOS, with deviations up to 1.35 mm buccolingually and 1.29 mm mesiodistally; digital impressions did not accurately reproduce soft tissue contours after ISFIR removal	Small sample size; pilot design; only single implants in the anterior maxilla; absence of repeated measurements; only linear (2D) measurements performed; short observation period
Ling et al. [12] prospective clinical pilot study	14 / 20 anterior maxillaIntervention: comparison of direct versus indirect IOS techniques for duplicating peri-implant soft tissue contour during definitive crown delivery; STL superimposition before and after delivery	TRIOS 3 (3Shape, Copenhagen, Denmark)Software: DentalDB (Exocad GmbH, Darmstadt, Germany)	Linear deviations of peri-implant mucosa (labial and palatal gingival margin height, thickness); volumetric deviations of labial and palatal soft tissue; PESComparator: direct digital impression technique based on peri-implant soft tissue profile immediately after provisional crown removal	Indirect technique resulted in significantly smaller labial and palatal tissue thickness deviations and lower labial soft-tissue volume changes compared with the direct technique; palatal tissue collapse occurred mainly with the direct technique; no significant differences in PES between techniques	Pilot design; limited sample size; no conventional impression control; short-term evaluation only; limited to single implants on only anterior maxilla; long-term esthetic stability not assessed
Docampo-Vázquez et al. [13] pilot clinical trial	10 / 10 mandibleIntervention: free gingival graft performed to increase peri-implant keratinized tissue; serial digital IOS obtained preoperatively and at 1 week, 1, 3, and 6 months to assess healing	True Definition (3M ESPE, Saint Paul, USA)Software: 3D Geomagic Capture Wrap (3D Systems, Rock Hill, USA)	Volumetric changes (mm³) of peri-implant soft tissues at donor and recipient sites; linear width of keratinized tissue; repeatability and reproducibility (Gage R&R)Comparator: conventional clinical linear measurements (endodontic file with rubber stop)	Digital technique demonstrated high repeatability and reproducibility (<10% variability); recipient sites showed significantly higher soft-tissue volume than donor sites at all follow-ups; no significant differences between digital and conventional methods for linear width, but conventional measurements lacked repeatability	Pilot study with small sample size; limited to implants in overdenture patients; no comparison with other soft-tissue augmentation techniques or materials; volumetric assessment limited to implant sites only

2

**Table 3:** Table Risk of bias assessment of the included studies

Study	Confounding	Selection of Participants	Classification of Intervention	Deviations from Intended Intervention	Missing Data	Measurement of Outcomes	Selection of Reported Results	Overall
Xiong et al. [9]	⨂	⊕	⊕	⊕	⊕	⨂	⊕	⨂
Silva Marques et al. [10]	⨂	⨂	⊕	⊕	⊕	⨂	⊕	⨂
Duran et al. [11]	⊝	⨂	⊕	⊕	⊕	⨂	⊕	⊝
Ling et al. [12]	⨂	⊕	⊕	⊕	⊕	⨂	⊕	⨂
Docampo-Vázquez et al. [13]	⊝	⨂	⊕	⊕	⊕	⊕	⊕	⊝
			⊕ Low	⨂ Unclear	⊝ High			

3

## Data Availability

Data available on request from the authors.
